# Machine Learning Models for Classifying Physical Activity in Free-Living Preschool Children

**DOI:** 10.3390/s20164364

**Published:** 2020-08-05

**Authors:** Matthew N. Ahmadi, Toby G. Pavey, Stewart G. Trost

**Affiliations:** 1 Institute of Health and Biomedical Innovation at Queensland Centre for Children’s Health Research, Queensland University of Technology, South Brisbane 4101, Australia; matthewnguyen.ahmadi@hdr.qut.edu.au; 2 Faculty of Health, School of Exercise and Nutrition Sciences, Queensland University of Technology, Kelvin Grove 4059, Australia; toby.pavey@qut.edu.au

**Keywords:** physical activity, accelerometer, measurement, supervised learning, classification, assessment, early childhood

## Abstract

Machine learning (ML) activity classification models trained on laboratory-based activity trials exhibit low accuracy under free-living conditions. Training new models on free-living accelerometer data, reducing the number of prediction windows comprised of multiple activity types by using shorter windows, including temporal features such as standard deviation in lag and lead windows, and using multiple sensors may improve the classification accuracy under free-living conditions. The objective of this study was to evaluate the accuracy of Random Forest (RF) activity classification models for preschool-aged children trained on free-living accelerometer data. Thirty-one children (mean age = 4.0 ± 0.9 years) completed a 20 min free-play session while wearing an accelerometer on their right hip and non-dominant wrist. Video-based direct observation was used to categorize the children’s movement behaviors into five activity classes. The models were trained using prediction windows of 1, 5, 10, and 15 s, with and without temporal features. The models were evaluated using leave-one-subject-out-cross-validation. The F-scores improved as the window size increased from 1 to 15 s (62.6%–86.4%), with only minimal improvements beyond the 10 s windows. The inclusion of temporal features increased the accuracy, mainly for the wrist classification models, by an average of 6.2 percentage points. The hip and combined hip and wrist classification models provided comparable accuracy; however, both the models outperformed the models trained on wrist data by 7.9 to 8.2 percentage points. RF activity classification models trained with free-living accelerometer data provide accurate recognition of young children’s movement behaviors under real-world conditions.

## 1. Introduction

Childhood obesity continues to be a serious global public health problem. In 2016, more than 41 million children between 0 and 5 years were overweight or obese [1]. The high prevalence of overweight and obesity among young children is cause for concern. Preschool-aged children who are overweight or obese are at an increased risk of type 2 diabetes, cardiovascular disease, and mental health problems as they progress to pre-adolescence and adulthood [2,3]. Childhood obesity is the top contributor to health care cost across all decades of life, with a 3 to 5 times higher health-care cost burden in adults with a history of childhood obesity [4].

Physical inactivity is a modifiable risk factor that contributes to the increase in the prevalence of overweight and obesity observed in young children [5,6]. Consequently, public health authorities have identified the preschool years as a critical period to intervene and promote regular physical activity [7,8,9]. The accurate measurement of physical activity is essential in order to identify and understand the individual, environmental and sociocultural determinants of physical activity, and to evaluate the effectiveness of physical activity intervention programs. Due to their unobtrusive size, robustness, and low cost, accelerometer-based motion sensors have become the method of choice for measuring physical activity in studies involving preschool-aged children [10].

Traditionally, cut-point methods have been used to classify physical activity intensity and estimate the time spent sedentary and in light, moderate, and vigorous physical activity. With this approach, the relationship between the measured energy expenditure and accelerometer counts is established using linear regression and thresholds or “cut-points” denoting the dividing line between sedentary-and-light (1.5 Metabolic Equivalents (METs)), light-and-moderate (3–4 METs), and moderate-and-vigorous physical activity (6 METs). Another common cut-point approach is the use of receiver operating characteristic (ROC) curves to determine the count threshold that provides the optimal combination of sensitivity and specificity for distinguishing between adjacent levels of physical activity intensity [11,12]. However, cut-point methods have been shown to have high misclassification rates among children. Validation studies involving independent samples of children indicate that cut-point approaches misclassify the true intensity of physical activity 35% to 45% of the time [13,14,15,16].

An alternative to cut-point methods is pattern recognition approaches, such as machine learning. Pattern recognition is a branch of artificial intelligence concerned with classifying or describing observations, with the goal of predicting outcomes based on previous knowledge or recognizable features in the raw data [17,18,19]. Accelerometer data processing techniques based on pattern recognition have been shown to provide accurate predictions of physical activity type and more accurate assessments of physical activity intensity [20,21,22]. Nonetheless, the uptake of machine learning methods by physical activity researchers has been slow, in part due to the difficulties of implementation, and the consistent finding that models trained on accelerometer data from laboratory-based activity trials do not generalize well to free-living environments [23,24,25].

We recently evaluated the accuracy of laboratory-trained machine learning activity classification models for preschool-aged children under true free-living conditions [26]. The models classified children’s physical activity behaviors activities into one of five activity classes: sedentary, light activities and games, moderate to vigorous activities and games, walk, and run. Under laboratory conditions, the overall classification accuracy ranged between 80% and 82% for models trained on hip data and 78% to 81% for models trained on wrist accelerometer data. However, when evaluated under true free-living conditions, the overall accuracy decreased by 10 to 20 percentage points to 66% to 70% for the hip models and 59% to 60% for the wrist models. Notably, there were substantial decreases in the walking recognition accuracy, which ranged from just 8% to 11% for the hip and 12% to 15% for the wrist. The reductions in classification accuracy under free-living conditions were attributed to several important methodological limitations. First, the laboratory-based models were trained using accelerometer data from a limited number of activities performed in a standardized manner. Under free-living conditions, a wide range of activities are performed, and physical activity behavior is far more variable. Second, the 15 s prediction window employed by the models may have been too long to capture the pulsatile and sporadic activity behaviors of preschool-aged children, resulting in prediction windows comprised of multiple activity types that are more difficult to classify. Third, the predictions for each activity window were made without considering temporal features, such as the variability in accelerometer signal in the preceding and succeeding activity windows. Incorporating information from the lag and lead windows may reduce noise and improve the classification of physical activity sequences. Fourth and finally, the classification models did not utilize features from multiple sensor placements through feature fusion. Models trained on data from multiple accelerometers placements have the potential to mitigate the weaknesses a single monitor placement may have for the detection of certain activities, such as activities with extensive upper body movement. While the aforementioned limitations of laboratory-based models have been reported previously [26], to our knowledge no previous study has developed activity classification models for preschool children trained exclusively on free-living data.

With this in mind, the current study evaluated the accuracy of machine learning activity classification models for preschool-aged children trained on true free-living accelerometer data. To address the limitations identified in our recent evaluation of laboratory-trained models, the classifiers were trained over a range of window sizes ranging from 1 to 15 s, the classification accuracy was evaluated with and without the inclusion of temporal features, and the models based on multiple accelerometer placements and feature fusion were evaluated. We hypothesized that the use of smaller prediction windows, the inclusion of temporal features, and feature fusion from multiple accelerometer placements would increase the classification performance under free-living conditions.

## 2. Materials and Methods

### 2.1. Participants

A total of 31 children between the ages of 3 and 5 years participated in the study. The sample was comprised of 9 girls and 22 boys, and there were approximately 10 children in each age category [26]. The children were recruited through a university email list-serv, local media, and word of mouth. Written parental consent was obtained prior to participation. The study was approved by the Queensland University of Technology’s Human Research Ethics Committee (approval number: 1700000423; date of approval—July 14, 2017).

### 2.2. Free-Living Play Session

Each child completed a 20 min active free play session at a location chosen by their parent or guardian. The locations that were chosen included the family home, community parks, and local green spaces [26]. The research team provided age-appropriate toys and play equipment, and the children were free to engage in any activity they desired. This allowed for natural activity behavior, transitions, and engagement with peers and the environment. The children were video recorded during the free play sessions with a hand-held Go-Pro Hero 5 (GoPro, Inc., San Mateo, CA, USA) camera for subsequent direct observation coding. Prior to each play session, an external timepiece was synchronized with the laptop computer used to initialize the accelerometers and displayed in front of the camera to ensure synchronization between the Go-Pro video files and accelerometer timestamps [26].

### 2.3. Instrumentation

During each free play session, the children wore an ActiGraph GT3X+ accelerometer (ActiGraph Corporation; Pensacola, FL, USA). The ActiGraph GT3X+ is a small and lightweight monitor that measures acceleration along three orthogonal axes with a dynamic range between +/− 6 g and a sampling frequency between 30 and 100 Hz. For the current study, the sampling frequency was set to 100 Hz. The ActiGraph monitors were worn on the children’s right hip and non-dominant wrist. For the hip location, the accelerometer was positioned on the right mid-axilla line at the level of the iliac crest. For the wrist location, the accelerometer was positioned on the posterior side of the arm, between the radial and ulnar styloid processes.

### 2.4. Direct Observation Coding Procedure

Go-Pro video files were imported into the Noldus Observer XT 14 software (Noldus Information Technology, Wageningen, The Netherlands) for continuous direct observation coding. A customized direct observation scheme was implemented in which the participant’s movement behavior was coded as one of the five activity classes predicted by the activity classification models [26]: sedentary (SED), light activities and games (LIGHT_AG), moderate-vigorous activities and games (MV_AG), walking (WALK), and running (RUN). A description of the activity classes is provided in Table 1. If a participant was not in view of the camera, movement behavior was coded as “out of view”. The computerized direct observation system generated a vector of date-time stamps corresponding to the start and finish of each movement event, which were used to calculate the event duration and assign the activity codes to the corresponding time segments of the accelerometer data. The inter-observer reliability was assessed by having two researchers independently code five randomly selected videos. Cohen’s unweighted kappa statistic for activity class was 0.86 (95% CI: 0.84–0.88), which, according to the ratings suggested by Landis and Koch [27], is almost perfect agreement.

### 2.5. Development and Evaluation of Activity Classification Models

#### 2.5.1. Data Processing and Feature Extraction

The annotated accelerometer data were segmented into non-overlapping sliding windows of 1, 5, 10, and 15 s. If the window contained multiple activity class codes (i.e., a combination of walking and running), the assigned code represented the activity class completed for the majority of the window (>50% window duration). To determine the impact of these “mixed windows” on the classifier performance, an indicator variable was created to flag windows with multiple activity codes. This enabled us to determine if the window size influenced the classifier performance by reducing the number of mixed prediction windows. Within each window, the tri-axial accelerometer signal was transformed into a single-dimension vector magnitude (VM), and two sets of features were extracted: the VM was selected to match the laboratory-based classification models and reduce the dimensionality.

(1) Base features: time and frequency domain features were used in the previously published activity classification models [21,22,28]: mean, SD, minimum, maximum, interquartile range, percentiles (10th, 25th, 50th, 75th, 95th), coefficient of variation, signal sum, signal power, peak-to-peak amplitude, median crossings, cross axis correlations, dominant frequency between 0.25 and 5.0 Hz, and magnitude of dominant frequency between 0.25 and 5.0 Hz.
(2) Base plus temporal features: a second feature set consisted of the base features and temporal features calculated from the preceding (lead) and succeeding (lag) activity windows. These included the standard deviation (SD) for the 1 and 2 lag and lead windows and the SD over the lag and lead windows and the current window ([$\sigma \;=\frac{1}{N}\sum_{i=0}^{N}{\left(x_{i}-\mu \right)}^{2}$], where *n* = 5). This resulted in a total of five temporal features, in addition to the base features.

#### 2.5.2. Model Training and Testing

Random Forest (RF) classification models were developed for each placement. An RF model was chosen because: (1) it is a widely implemented ensemble-based supervised learning algorithm that has been shown to provide robust results in prior activity recognition studies [29]; (2) it requires minimal data pre-processing, as the features do not need to be normalized; and (3) feature selection procedures are not required because the algorithm effectively does this on its own [30]. The models were implemented using the “randomForest” and “caret” packages within R software. The number of features randomly sampled at each node (tuning parameter mtry) was optimized during training, whilst the number of trees was kept constant at 500. The fully annotated training datasets, r scripts for the final RF models, and data required to run the classification models are available at the link provided in the Supplementary Materials section.

The out of sample prediction error was evaluated using leave-one-subject-out-cross-validation (LOSO-CV). In LOSO-CV, the model is trained on data from all the participants except one, which is “held out” and used as the test data set. The process is repeated until all the participants have served as the test data, and the performance metrics are aggregated [31]. F-scores were used to assess the accuracy of each model. F-scores were calculated because they is based on the harmonic mean of precision and recall and are less biased by class size imbalances [32]. The F-scores were calculated for each activity class and averaged to provide an overall F-score for each classifier. Additionally, to identify patterns of misclassification, confusion matrices were generated for each model.

### 2.6. Statistical Analysis

A 3 × 2 × 4 repeated measures ANOVA was run to examine the effects of placement (wrist, hip, combined wrist and hip), feature set (base vs. temporal features), and window size (1, 5, 10, and 15 s) on the F-scores. The significant main effects and interactions were evaluated using tests of simple effects and pre-planned single degree freedom contrasts. Statistical significance was set at an alpha level of 0.05.

## 3. Results

The results of the repeated measures ANOVA identified a significant two-way feature set by window size interaction (F _3,90_ = 3.40, *p* = 0.02), indicating that the effects of the feature set on the F-scores differed by window size. The two-way placement by feature set interaction (F _2,60_ = 1.88, *p* = 0.17), placement by window size interaction (F _6,180_ = 0.79, *p* = 0.58), and three-way placement by feature set by window size interaction (F _6,180_ = 0.20, *p* = 0.97) were not statistically significant.

**Figure 1:**
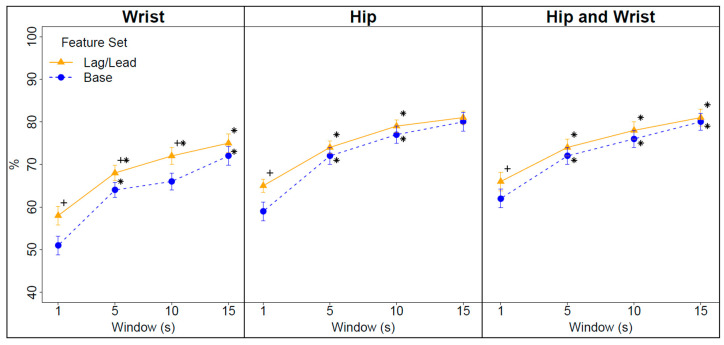
Interaction plots summarizing the effect of window size and feature set on the adjusted F-scores for models trained on wrist, hip, and combined hip and wrist accelerometer data. + Denotes significantly different from the base model at a given window size *p* < 0.05; * Denotes significantly different from the previous window size for a given feature set *p* < 0.05.

Figure 1 displays interaction plots summarizing the effects of window size and feature set on the F-scores at each accelerometer placement. The numbers of prediction intervals with multiple activity types (mixed windows) for models trained on 1, 5, 10, and 15 s windows were 3527 (9.2%), 2255 (29.5%), 1597 (42.1%), and 1258 (50.2%), respectively. For the wrist and combined hip and wrist models, the F-scores increased significantly as the window size increased from 1 to 15 s. However, for the models trained on hip data, the F-scores failed to improve significantly as the window size increased from 10 to 15 s.

For the models trained on wrist data on 1, 5, and 10 s windows and the models trained on the hip and combined hip and wrist data on 1 s windows, the addition of lag/lead features resulted in small but significant improvements in the F-scores. For the wrist model trained on 15 s windows and the hip and combined hip and wrist models trained on 5, 10, and 15 s windows, the addition of lag/lead features did not significantly improve the performance.

Across all window sizes and for both feature sets, the classification models trained on the wrist accelerometer data exhibited significantly lower F-scores than the models trained on the hip or combined hip and wrist accelerometer data. On average, the performance differential was 7.9 to 8.2 percentage points. There were no significant differences in the F-scores between the models trained on the hip accelerometer data and the combined hip and wrist accelerometer data.

The F-scores for the five activity classes and the weighted average F-score for each model are reported in Table 2. The class-level F-scores tended to increase as the window size increased from 1 to 15 s. The inclusion of lag/lead features resulted in marginal increases in the F-scores; however, this was not true for all models, particularly for the WALK and RUN activity classes. Only the lag/lead wrist model trained on 10 s windows and the hip and combined hip and wrist models trained on the 10 and 15 s windows exhibited an average F-score of ≥ 80% and provided F-scores of ≥ 70% for all five activity classes. The wrist lag/lead model trained on 15 s windows achieved an average F-score of ≥ 80%; however, the F-score for WALK was just under 70%.

**Figure 2:**
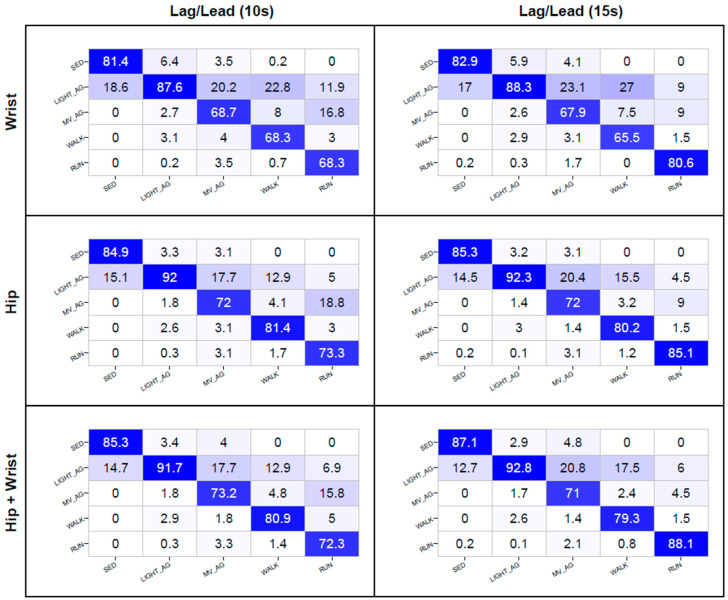
Confusion matrices for physical activity classification from the wrist, hip, and combined hip and wrist placement for lag/lead 10 and 15 s window models. The columns represent observed; rows represent predictions; bold represents correct predictions; SED = sedentary; LIGHT_AG = light physical activity and games; MV_AG = moderate to vigorous physical activity and games; WALK = walking; RUN = running.

Heat map confusion matrices for the wrist, hip, and combined hip and wrist classifiers are presented in Figure 2. To reduce the complexity, only the results for the lag/lead models trained on 10 and 15 s windows are reported. Detailed confusion matrices for all 24 classification models can be found in the Supplementary Files.

For the wrist placement, the lag/lead 10 and 15 s window models’ recognition of SED (81.4%–82.9%) and LIGHT_AG (87.6%–88.3%) were good. The SED instances were most frequently misclassified as LIGHT_AG (17.0%–18.6%), while a small percentage of LIGHT_AG instances were misclassified as either SED (5.9%–6.4%), MV_AG (2.6%–2.7%), or WALK (2.9%–3.1%). The recognition of RUN was also good for the 15 s model (80.6%), but only modest for the 10 s model (68.3%). For the 15 s model, RUN was most frequently misclassified as either LIGHT_AG (9.0%–11.9%) or MV_AG (9.0%–16.8%), with only a very small percentage of the instances misclassified as WALK (1.5%–3.0%). The recognition of MV_AG (67.9%–68.7%) and WALK (65.5%–68.3%) was modest, with approximately 20.0% to 27.0% of MV_AG and WALK instances misclassified as LIGHT_AG.

For the hip placement, the lag/lead 10 and 15 s window models’ recognitions were good to excellent for SED and LIGHT_AG (84.9% to 92.3%). The majority of SED instances were misclassified as LIGHT_AG (14.5%–15.1%), while a small percentage of LIGHT_AG was misclassified as either SED (3.2%–3.3%), WALK (2.6%–3.0%), or MV_AG (1.4%–1.8%). The recognition of RUN was good for the 15 s window models (85.1%) and acceptable for the 10 s windows (73.3%). RUN was most frequently misclassified as MV_AG (9.0%–18.8%). The recognition of WALK was good for the 10 and 15 s windows (80.2%–81.4%). WALK was most frequently misclassified as LIGHT_AG (12.9%–15.5%). The recognition of MV_AG was acceptable (72.0%), with 17.7% to 20.4% of instances misclassified as LIGHT_AG.

For the combined hip and wrist classifiers, the 10 s window, and the 15 s window models, the recognition was good to excellent for SED and LIGHT_AG (85.3%–92.8%). Almost all the misclassifications of SED instances were as LIGHT_AG (12.7%–14.7%), and a small percentage of LIGHT_AG was misclassified as either SED (2.9%–3.4%), WALK (2.6%–2.9%), or MV_AG (1.7%–1.8%). The recognition of RUN was good for the 15 s window model (88.1%), and acceptable for the 10 s window model (72.3%). For the 10 s window model, RUN was most frequently misclassified as MV_AG (15.8%), whilst for 15 s window model, misclassification occurred mostly as either LIGHT_AG (4.5%) or MV_AG (6.0%). The recognition of WALK was good for the 10 s and 15 s window models (79.3%–80.9%). WALK was most frequently misclassified as LIGHT_AG (12.9%–17.5%). The recognition of MV_AG was acceptable (71.0%–73.2%), with 17.7% to 20.8% of instances misclassified as LIGHT_AG.

## 4. Discussion

Machine learning approaches to accelerometer data processing have emerged as more versatile and potentially more accurate alternative to cut-point methods. However, when laboratory-trained activity classification models are evaluated under free-living conditions, substantial decreases in accuracy have been reported [23,25,33]. In the current study, the preschooler activity classification models trained on free-living accelerometer data exhibited a substantially higher accuracy than that reported for comparable models trained on laboratory data. Under free-living conditions, F-scores for the best performing wrist and hip model were 80.6% and 85.9%, respectively. In comparison, F-scores for the corresponding laboratory trained model was 60.2% for the wrist and 64.4% for the hip [26]. In contrast to the poor detection of walking reported for the laboratory-trained model, which ranged between just 8% and 15%, the free-living models correctly classified 68% to 82% of the walking instances.

Our findings are consistent with the results of adult studies comparing the performance of classification models trained with laboratory and free-living data under free-living conditions. Among older adults, Sasaki et al. [23] reported overall accuracies ranging from 49% to 55% for RF and support vector machine physical activity classification models trained on laboratory-based accelerometer data from the wrist, hip, or ankle. When the same models were trained on free-living data, the accuracy increased by 9–15 percentage points to 58% to 69%. Ermes et al. [33] trained decision tree and artificial neural network physical activity classification models using free-living data and observed an overall accuracy of 89% compared to 72% when the models were trained on laboratory data. Similarly, Bastian et al. [25] reported respective increases of 71 and 40 percentage points for the detection of sedentary activities and cycling after training a Bayesian activity classification model on both laboratory and free-living data. These findings, together with the results of the current study, provide consistent evidence that machine learning activity classification models should be trained and tested with data collected under true free-living conditions.

The choice of window size or epoch length has significant implications for the accurate assessment of physical activity in young children [34,35,36,37]. Several studies have noted that young children perform physical activities intermittently in short bursts lasting a few seconds [38,39]. Therefore, for classification models predicting specific physical activity classes or types, it is important that the prediction windows be as short as possible so that the number of windows containing multiple activities can be minimized. In this study, it was hypothesized that a smaller window would reduce the proportion of “mixed windows” and therefore improve the classification accuracy. To test this hypothesis, the models were trained using window sizes of 1, 5, 10, and 15 s. Contrary to our hypothesis, the F-scores increased as the window size increased. While shorter prediction intervals reduced the number of windows with multiple activity types, the costs of mixed activity windows were more than offset by the benefits of having sufficient information to reliably capture movement patterns. Of the 24 models tested, only the wrist lag/lead model trained on 10 s data, and the hip and combined hip and wrist models with prediction windows of 10 or 15 s achieved average F-scores of ≥ 80% and provided F-scores ≥ 70% for all five activity classes.

For all the accelerometer placements, the models trained on 1 s windows did not provide an acceptable accuracy. The inferior performance of these models was due, in large part, to the poor recognition of MV_AG, WALK, and RUN. Between 26% and 52% of the MV_AG and WALK instances were misclassified as LIGHT_AG, and between 24% and 39% of the RUN instances were misclassified as MV_AG. It was evident that a prediction window of only 1 s provided insufficient information to reliably differentiate between these activity classes. With just 1 s of data (100 instances), features that differentiated MV_AG and WALK from LIGHT_AG (e.g., log energy, dominant frequency magnitude, entropy) over longer prediction windows were similar in magnitude; likewise, features that differentiated RUN from MV_AG (e.g., mean absolute deviation, signal power, entropy) were similar in magnitude. With longer prediction windows, more data was accumulated, thereby increasing the precision with which discriminative features were calculated. Consequently, the detection accuracy increased for these activity classes.

In support of our hypothesis, the addition of features from lag and lead windows significantly improved the free-living classification accuracy. Temporal features have previously been used in studies developing and testing physical activity classification models, although their relative importance was not systematically evaluated. Zhang et al. [40] used the ratio of the dominant frequency recorded for the current and previous window to train decision tree and support vector machine activity classification models for the wrist and hip. For both accelerometer placements, the overall classification accuracy exceeded 95%, although the model was trained and tested using laboratory-based activity trials. Among preschool-aged children, Zhao et al. [41] used the lag and lead ActiGraph activity counts and step counts to train a support vector machine activity classifier. The overall accuracy exceeded 85%.

In the current study, the inclusion of temporal features based on the standard deviation of the signal vector magnitude was most beneficial for the models trained on the wrist accelerometer data. For these models, the inclusion of temporal features increased the overall F-score by an average of six percentage points, largely through the improved detection of SED, LIGHT_AG, and MV_AG. When performing physical activities in these three class categories, the accelerometer data recorded at the wrist exhibited significantly greater window-to-window variability than the accelerometer data recorded at the hip. Thus, for models trained on the wrist accelerometer data, the inclusion of temporal features related to signal variability in lag and lead windows was informative for classifying physical activities in these classes. Conversely, for clearly defined rhythmic activities such as walking and running, the temporal stability of the accelerometer signal was less dependent on the accelerometer placement.

Contrary to our hypothesis, the development of models based on multiple accelerometer placements and feature fusion did not improve the free-living classification performance. This could be a function of the simple feature fusion approach implemented in the current study. Feature fusion approaches that use a class-based decision fusion technique may better capitalize on the increased information and features resulting from multiple accelerometer placements [42]. Alternatively, the lack of improvement might be due to a less than optimal combination of accelerometer placements. Narayanan et al. [43] reported a greater overall accuracy for a physical activity classifier trained on accelerometer data from the thigh and lower back than classifiers trained on data from the wrist and back and the wrist and thigh.

When evaluated under laboratory conditions, activity classification models trained on hip and wrist accelerometer data exhibit comparable accuracy [22,44,45]. However, in the current study, the classifiers trained on the hip accelerometer data exhibited significantly higher F-scores than the wrist models. The discrepancy in findings may be attributable, in part, to differences in how the machine learning classification models are trained and tested in laboratory-based studies compared to free-living studies. When classification models are trained in the laboratory setting, the participants complete a small number of choreographed activity trials which do not replicate how activities are performed in free-living scenarios. Moreover, when models are trained on free-living accelerometer data, the participants complete entirely different activities or complete the same activities in an entirely different manner. Furthermore, laboratory-based studies typically do not include activity trials that test the limitations of wrist mounted accelerometers. Sedentary and light intensity activities tend to be performed with limited upper limb movements (e.g., sitting on the floor listening to story, standing, and painting), while walking and running trials are performed continuously with arms oscillating freely. Under true free-living conditions, the participants often walk or run intermittently while holding objects, resulting in limited arm movements. This presents unique challenges for wrist classifiers and could account for the lower classification accuracy observed for the wrist models under true free-living conditions.

The free-living activity classifiers developed and validated in the current study can be implemented in field-based studies involving preschool-aged children and offer several advantages over traditional cut-point methods. By providing physical activity metrics based on the combination of activity type and intensity, the classification models allow researchers to monitor not only the quantity of physical activity, but also the quality of movement behaviors. For example, the classifiers can be used to measure how time in active game play is accumulated at home or early childhood education and care settings. Further, the study provides sufficiently accurate activity classification models for wrist mounted accelerometers. Wrist-worn accelerometers provide a higher compliance than accelerometers worn on the hip and allows researchers to concurrently measure sleep and evaluate adherence to newly released 24-hour movement guidelines. Furthermore, by classifying the activity class categories rather than the physical activity intensity based on energy expenditure, the models provide investigators with a measurement tool to examine age-related trends in movement behavior that are not confounded by developmental differences in the energy cost of physical activity [46,47,48].

The current study had several strengths. It is the first study to develop and evaluate the accuracy of machine learning activity classification models for preschool-aged children trained on free-living data. Ground-truth activity was obtained using a rigorous video-based direct observation and a continuous coding procedure. In addition, the study evaluated accuracy across multiple window sizes, feature sets, and accelerometer placements. Furthermore, mixed windows, in which multiple activities were performed within a given window, were not excluded from the evaluation which enhances the ecological validity and generalizability of the findings.

Opposing these strengths were several limitations. First, due to the demands of the data collection protocol, the free-living play sessions were restricted to 20 min. Although the participants were free to engage in any activity they chose at any location, longer play sessions may have provided a greater variety of moderate-to-vigorous intensity activities and more prolonged bouts of walking and running instances to train the models. Second, the study had a modest sample size of 31 participants. Nevertheless, the free play sessions generated more than 3.8 million data points and provided between 2500 and 38,500 activity windows, which was sufficient to train and test the models. Third, for classification models achieving average F-scores ≥ 80%, between 13% and 30% of the observed MV_AG and WALK instances were misclassified as LIGHT_AG. The propensity for activity classifiers trained solely on triaxial accelerometer data to misclassify certain activities in young children such as slow intermittent walking, walking while holding objects, and climbing on fixed playground equipment has been noted previously [26]. Therefore, to improve the recognition of MV_AG and WALK, future studies should include features from additional sensors, such as heart rate monitors, gyroscopes, barometric pressure sensors, and GPS, which could provide additional information about physical activity intensity, posture, and changes in position and elevation. Fourth and finally, our study only trained RF classifiers and did not benchmark performance with other supervised learning algorithms. Because the focus of the current study was to evaluate activity classification models trained on true free-living data and compare the performance with models trained on laboratory data, it was necessary to employ the same supervised learning algorithm. Future studies could train and test classifiers using other machine learning algorithms and benchmark performance against the RF classifiers developed in the current study. The fully annotated training data for all window sizes are available at the following link: https://github.com/QUTcparg/PS_PAClassification.

## 5. Conclusions

In summary, machine learning activity classification models trained on free-living data for preschool-aged children exhibited an acceptable accuracy under free-living conditions. The random forest activity classifiers with prediction windows of 10 or 15 s provided the accurate recognition of five activity classes representative of young children’s movement behaviors. The inclusion of lag and lead features improved classification accuracy, with the largest increases observed for the wrist placement. The hip and combined hip and wrist classification models provided comparable accuracy, with both models outperforming the models trained on wrist accelerometer data. Future studies should train models using accelerometer data collected over extended time periods and a wider range of settings to provide more movement diversity in the training data. Such studies should explore the inclusion of additional temporal features, such as the ratio of the dominant frequency for the current and adjacent windows or information/features from additional sensors such as heart rate monitors, gyroscopes, barometric pressure sensors, and GPS trackers.

## Figures and Tables

**Table 1:** Description of the five activity classes.

Activity Class	Movement Descriptors	Activity Types
SED	Sitting/lying down	Sit still
	Stationary/motionless	Sit w/upper body movement
LIGHT_AG	Standing	Stand still
	Stationary/movement of limbs or trunk (very easy)	Stand w/upper body movement
	Translocation (slow/easy)	Crawl
		Up/downstairs
		Floor games
		Stand and kick
		Slide
		Climb (low intensity)
MV_AG	Translocation (medium speed/moderate)	Run and kick
	Translocation (fast or very fast/hard)	Side gallop
		Jump/hop/leap
		Ride a bike
		Ride a scooter
		Stationary ride/spin/swing
		Climb (high intensity)
WALK	Translocation (steady/medium speed/moderate)	Walk slow/stroll
		Walk brisk
		Walk and hold object
RUN	Translocation (steady/fast or very fast/hard)	Sprint
		Run and hold object

SED = sedentary; LIGHT AG = light activity and games; MV AG = moderate to vigorous activities and games; WALK = walking; RUN = running

**Table 2:** F-scores for the five activity classes and the weighted average F-score for each model.

Placement	Feature	Window	SED	LIGHT_AG	MV_AG	WALK	RUN	Ave F-Score
Wrist	Base	1	63.3	71.4	45.7	45.9	55.1	62.6
		5	69.3	76.5	60.9	60.7	68.5	70.8
		10	73.7	79.1	62.0	68.8	73.4	74.5
		15	78.2	81.2	62.1	70.5	82.4	77.3
	Lag/Lead	1	69.2	75.5	57.5	54.8	61.6	68.8
		5	78.5	80.1	66.9	60.3	68.8	75.5
		10	82.4	82.9	70.3	70.8	71.5	80.0
		15	83.3	83.7	70.7	69.0	82.4	80.6
Hip	Base	1	73.3	76.0	61.0	55.7	63.1	70.6
		5	80.6	82.7	75.5	69.8	71.2	79.5
		10	82.3	85.7	77.6	80.7	74.4	83.1
		15	85.0	86.8	75.3	78.4	80.0	84.0
	Lag/Lead	1	80.0	80.4	65.2	62.9	67.2	75.8
		5	85.7	85.6	76.3	68.8	72.8	82.2
		10	87.6	87.9	76.4	81.0	73.6	85.3
		15	87.7	88.2	78.5	79.4	82.6	85.9
Hip & Wrist	Base	1	75.8	77.7	62.1	58.4	64.5	72.5
		5	81.1	82.8	74.3	70.1	71.7	79.6
		10	83.9	85.8	75.6	79.5	74.9	83.2
		15	85.5	86.4	77.1	78.1	86.6	84.3
	Lag/Lead	1	80.8	81.0	66.7	64.9	67.9	76.8
		5	86.0	85.6	74.5	69.5	72.5	82.1
		10	87.5	87.9	77.2	80.7	73.0	85.3
		15	88.7	88.4	78.0	79.8	86.8	86.4

## Supplementary Materials

The annotated training datasets, final RF models, and sample data with r scripts to run the classification models are available at the following link: https://github.com/QUTcparg/PS_PAClassification.
