# Supplementary material for: Machine Learning Models for Classifying Physical Activity in Free-Living Preschool Children
Source: Sensors (Basel). 2020 Aug 5;20(16):4364. doi: 10.3390/s20164364 (PMC7472058; doi:10.3390/s20164364)
Supplement: Supplementary file 1 [file sensors-20-04364-s001.pdf]

Wrist Confusion Matrix

**Activity Class**	**1**	**2**	**3**	**4**	**5**	**1**	**2**	**3**	**4**	**5**
			Base (1 s)					Lag/Lead (1 s)		
1. SED	**56.8 [5008]**	42.4 [3741]	0.4 [33]	0.4 [32]	0.0 [0]	**64.9 [5716]**	35.0 [3082]	0.1 [13]	0.0 [3]	0.0 [0]
2. LIGHT_AG	9.2 [1731]	**81.1 [15269]**	5.8 [1086]	3.8 [721]	0.2 [31]	9.2 [1728]	**83.1 [15650]**	5.0 [939]	2.6 [499]	0.1 [22]
3. MV_AG	4.9 [262]	42.5 [2272]	**42.3 [2261]**	6.3 [339]	4.0 [214]	4.7 [254]	31.4 [1678]	**56.1 [3002]**	4.9 [263]	2.8 [151]
4. WALK	0.1 [6]	51.2 [2244]	10.4 [457]	**37.4 [1638]**	0.8 [34]	0.1 [3]	42.2 [1850]	12.5 [547]	**44.5 [1950]**	0.7 [29]
5. RUN	0.0 [0]	11.2 [122]	38.1 [414]	2.9 [32]	**47.7 [519]**	0.0 [0]	8.3 [90]	36.5 [397]	2.4 [26]	**52.8 [574]**
			Base (5 s)					Lag/Lead (5 s)		
1. SED	**65.3 [1142]**	34.3 [600]	0.3 [5]	0.1 [2]	0.0 [0]	**76.9 [1345]**	23.0 [402]	0.1 [1]	0.1 [1]	0.0 [0]
2. LIGHT_AG	9.4 [358]	**82.6 [3161]**	4.5 [173]	3.3 [128]	0.1 [5]	7.5 [285]	**85.4 [3268]**	3.6 [138]	3.4 [129]	0.1 [5]
3. MV_AG	4.7 [47]	29.0 [288]	**59.8 [592]**	4.0 [40]	2.3 [23]	4.8 [48]	22.3 [221]	**66.5 [658]**	3.9 [39]	2.4 [24]
4. WALK	0.1 [1]	37.3 [325]	10.0 [87]	**52.3 [456]**	0.3 [3]	0.0 [0]	39.0 [340]	8.6 [75]	**51.7 [451]**	0.7 [6]
5. RUN	0.0 [0]	6.2 [13]	32.1 [67]	1.9 [4]	**59.8 [125]**	0.0 [0]	6.7 [14]	29.7 [62]	2.4 [5]	**61.2 [128]**
			Base (10 s)					Lag/Lead (10 s)		
1. SED	**72.6 [633]**	27.3 [238]	0.0 [1]	0.0 [0]	0.0 [0]	**81.4 [710]**	18.6 [162]	0.0 [0]	0.0 [0]	0.0 [0]
2. LIGHT_AG	9.5 [186]	**83.6 [1633]**	3.6 [70]	3.1 [60]	0.2 [4]	6.4 [125]	**87.6 [1711]**	2.7 [53]	3.1 [60]	0.2 [4]
3. MV_AG	6.0 [27]	23.0 [104]	**61.5 [278]**	6.0 [27]	3.5 [16]	3.5 [16]	20.2 [91]	**68.7 [311]**	4.0 [18]	3.5 [16]
4. WALK	0.0 [0]	21.5 [89]	11.1 [46]	**66.7 [277]**	0.7 [3]	0.2 [1]	22.8 [94]	8.0 [33]	**68.3 [284]**	0.7 [3]
5. RUN	0.0 [0]	5.0 [5]	22.8 [23]	1.0 [1]	**71.3 [72]**	0.0 [0]	11.9 [12]	16.8 [17]	3.0 [3]	**68.3 [69]**
			Base (15 s)					Lag/Lead (15 s)		
1. SED	**78.6 [445]**	21.2 [120]	0.0 [0]	0.0 [0]	0.2 [1]	**82.9 [469]**	17.0 [96]	0.0 [0]	0.0 [0]	0.2 [1]
2. LIGHT_AG	8.3 [111]	**84.7 [1128]**	3.7 [49]	3.0 [40]	0.2 [3]	5.9 [79]	**88.3 [1175]**	2.6 [35]	2.9 [38]	0.3 [4]
3. MV_AG	5.5 [16]	29.5 [85]	**59.5 [173]**	3.4 [10]	2.1 [6]	4.1 [12]	23.1 [67]	**67.9 [197]**	3.1 [9]	1.7 [5]
4. WALK	0.0 [0]	22.0 [56]	9.9 [25]	**68.1 [171]**	0.0 [0]	0.0 [0]	27.0 [68]	7.5 [19]	**65.5 [165]**	0.0 [0]
5. RUN	0.0 [0]	6.0 [4]	13.4 [9]	0.0 [0]	**80.6 [54]**	0.0 [0]	9.0 [6]	9.0 [6]	1.5 [1]	**80.6 [54]**

Numbers represent: % [# of instances]

Hip Confusion Matrix

Activity Class	1	2	3	4	5	1	2	3	4	5
	Base (1 s) (71.0%)					Lag/Lead (1 s) (76.1%)				
1. SED	**70.2 [6191]**	29.3 [2582]	0.4 [31]	0.1 [10]	0.0 [0]	**78.3 [6905]**	21.5 [1892]	0.2 [15]	0.0 [2]	0.0 [0]
2. LIGHT_AG	9.6 [1814]	**80.3 [15136]**	4.7 [888]	5.0 [950]	0.3 [50]	8.0 [1516]	**84.2 [15860]**	4.4 [829]	3.1 [589]	0.2 [44]
3. MV_AG	0.9 [48]	30.5 [1631]	**57.6 [3080]**	6.9 [369]	4.1 [266]	0.6 [30]	26.8 [1432]	**63.3 [3387]**	6.3 [338]	3.0 [161]
4. WALK	0.2 [9]	36.1 [1580]	11.3 [495]	**51.0 [2232]**	1.4 [81]	0.0 [0]	30.9 [1354]	11.9 [522]	**56.1 [2455]**	1.1 [48]
5. RUN	0.3 [3]	7.6 [83]	24.5 [266]	7.4 [81]	**60.2 [654]**	0.0 [0]	6.3 [68]	26.7 [290]	4.7 [51]	**62.4 [678]**
	Base (5 s) (79.7%)					Lag/Lead (5 s) (82.4%)				
1. SED	**79.5 [1390]**	20.4 [356]	0.1 [1]	0.1 [2]	0.0 [0]	**81.9 [1433]**	18.0 [315]	0.0 [0]	0.1 [1]	0.0 [0]
2. LIGHT_AG	7.9 [303]	**85.6 [3276]**	2.7 [104]	3.5 [133]	0.2 [9]	3.9 [150]	**90.2 [3450]**	2.5 [95]	3.2 [121]	0.2 [9]
3. MV_AG	0.5 [5]	20.6 [204]	**72.0 [713]**	3.3 [33]	3.5 [35]	1.4 [14]	19.5 [193]	**72.5 [718]**	3.4 [34]	3.1 [31]
4. WALK	0.0 [0]	28.4 [248]	5.4 [47]	**64.7 [564]**	1.5 [13]	0.0 [0]	30.5 [266]	5.2 [45]	**62.6 [546]**	1.7 [15]
5. RUN	0.0 [0]	7.7 [16]	15.8 [33]	6.2 [13]	**70.3 [147]**	0.0 [0]	5.7 [12]	16.3 [34]	5.7 [12]	**72.2 [151]**
	Base (10 s) (84.1%)					Lag/Lead (10 s) (86.4%)				
1. SED	**81.0 [706]**	19.0 [166]	0.0 [0]	0.0 [0]	0.0 [0]	**84.9 [740]**	15.1 [132]	0.0 [0]	0.0 [0]	0.0 [0]
2. LIGHT_AG	6.3 [123]	**88.8 [1735]**	1.9 [37]	2.8 [55]	0.2 [3]	3.3 [64]	**92.0 [1797]**	1.8 [36]	2.6 [51]	0.3 [5]
3. MV_AG	1.1 [5]	18.1 [83]	**73.7 [333]**	3.5 [16]	3.5 [16]	3.1 [14]	17.7 [81]	**72.0 [329]**	3.1 [14]	3.1 [14]
4. WALK	0.0 [0]	11.2 [47]	4.3 [18]	**82.0 [340]**	2.4 [10]	0.0 [0]	12.9 [53]	4.1 [17]	**81.4 [338]**	1.7 [7]
5. RUN	0.0 [0]	5.0 [5]	15.8 [16]	3.0 [3]	**76.2 [77]**	0.0 [0]	5.0 [5]	18.8 [19]	3.0 [3]	**73.3 [74]**
	Base (15 s) (85.1%)					Lag/Lead (15 s) (87.0%)				
1. SED	**82.9 [469]**	17.0 [96]	0.0 [0]	0.0 [0]	0.2 [1]	**85.3 [483]**	14.5 [82]	0.0 [0]	0.0 [0]	0.2 [1]
2. LIGHT_AG	4.6 [61]	**90.7 [1207]**	1.7 [23]	2.9 [39]	0.1 [1]	3.2 [43]	**92.3 [1229]**	1.4 [18]	3.0 [40]	0.1 [1]
3. MV_AG	2.8 [8]	21.1 [62]	**69.9 [202]**	2.1 [6]	4.1 [12]	3.1 [9]	20.4 [59]	**72.0 [209]**	1.4 [4]	3.1 [9]
4. WALK	0.0 [0]	14.9 [37]	5.6 [14]	**78.4 [198]**	1.2 [3]	0.0 [0]	15.5 [39]	3.2 [8]	**80.2 [202]**	1.2 [3]
5. RUN	0.0 [0]	6.0 [4]	10.4 [7]	0.0 [0]	**83.6 [56]**	0.0 [0]	4.5 [3]	9.0 [6]	1.5 [1]	**85.1 [57]**

Numbers represent: % [# of instances]

Hip and Wrist Confusion Matrix

Activity Class	1	2	3	4	5	1	2	3	4	5
	Baseline (1 s) (72.9%)					Lag/Lead (1 s) (77.1%)				
1. SED	**71.9 [6334]**	27.9 [2456]	0.2 [22]	0.0 [2]	0.0 [0]	**78.4 [6909]**	21.5 [1895]	0.1 [10]	0.0 [0]	0.0 [0]
2. LIGHT_AG	8.1 [1517]	**83.0 [15636]**	4.6 [867]	4.1 [778]	0.2 [40]	7.1 [1340]	**85.5 [16103]**	4.2 [793]	3.0 [569]	0.2 [33]
3. MV_AG	0.8 [45]	30.7 [1642]	**58.8 [3142]**	6.1 [328]	3.6 [191]	0.6 [31]	27.5 [1472]	**64.6 [3455]**	4.5 [239]	2.8 [151]
4. WALK	0.2 [7]	36.7 [1607]	9.9 [433]	**52.2 [2288]**	1.0 [44]	0.0 [1]	31.3 [1370]	10.4 [456]	**57.4 [2513]**	0.9 [39]
5. RUN	0.2 [2]	7.5 [81]	27.7 [301]	5.1 [55]	**59.6 [648]**	0.0 [0]	6.6 [72]	27.3 [297]	4.0 [44]	**62.0 [674]**
	Baseline (5 s) (79.8%)					Lag/Lead (5 s) (82.3%)				
1. SED	**78.8 [1379]**	21.1 [369]	0.1 [1]	0.0 [0]	0.0 [0]	**83.0 [1451]**	17.0 [298]	0.0 [0]	0.0 [0]	0.0 [0]
2. LIGHT_AG	7.0 [268]	**86.5 [3307]**	3.1 [118]	3.3 [125]	0.2 [7]	4.0 [154]	**89.9 [3437]**	2.6 [99]	3.4 [130]	0.1 [5]
3. MV_AG	0.4 [4]	22.0 [218]	**71.6 [709]**	2.7 [27]	3.2 [32]	2.0 [20]	20.6 [204]	**70.9 [702]**	2.8 [28]	3.6 [36]
4. WALK	0.0 [0]	28.8 [251]	5.8 [51]	**63.9 [557]**	1.5 [13]	0.0 [0]	29.8 [260]	5.8 [51]	**63.5 [554]**	0.8 [7]
5. RUN	0.0 [0]	7.7 [16]	18.7 [39]	3.8 [8]	**69.9 [146]**	0.0 [0]	5.3 [11]	20.1 [42]	4.8 [10]	**69.9 [146]**
	Baseline (10 s) (84.1%)					Lag/Lead (10 s) (86.3%)				
1. SED	**82.3 [718]**	17.7 [154]	0.0 [0]	0.0 [0]	0.0 [0]	**85.3 [744]**	14.7 [128]	0.0 [0]	0.0 [0]	0.0 [0]
2. LIGHT_AG	5.7 [111]	**88.9 [1737]**	2.1 [41]	3.1 [60]	0.2 [4]	3.4 [66]	**91.7 [1790]**	1.8 [36]	2.9 [56]	0.3 [5]
3. MV_AG	2.4 [11]	19.2 [87]	**71.9 [325]**	2.9 [13]	3.5 [16]	4.0 [18]	17.7 [80]	**73.2 [331]**	1.8 [8]	3.3 [15]
4. WALK	0.0 [0]	13.0 [54]	5.3 [22]	**80.3 [333]**	1.4 [6]	0.0 [0]	12.9 [53]	4.8 [20]	**80.9 [336]**	1.4 [6]
5. RUN	0.0 [0]	4.0 [4]	17.8 [18]	3.0 [3]	**75.2 [76]**	0.0 [0]	6.9 [7]	15.8 [16]	5.0 [5]	**72.3 [73]**
	Baseline (15 s) (85.4%)					Lag/Lead (15 s) (87.5%)				
1. SED	**83.9 [475]**	15.9 [90]	0.0 [0]	0.0 [0]	0.2 [1]	**87.1 [493]**	12.7 [72]	0.0 [0]	0.0 [0]	0.2 [1]
2. LIGHT_AG	4.7 [62]	**90.4 [1203]**	1.8 [24]	3.2 [42]	0.0 [0]	2.9 [39]	**92.8 [1235]**	1.7 [22]	2.6 [34]	0.1 [1]
3. MV_AG	2.8 [8]	23.2 [67]	**71.0 [206]**	1.0 [3]	2.1 [6]	4.8 [14]	20.8 [60]	**71.0 [206]**	1.4 [4]	2.1 [6]
4. WALK	0.0 [0]	18.0 [45]	2.8 [7]	**78.4 [198]**	0.8 [2]	0.0 [0]	17.5 [44]	2.4 [6]	**79.3 [200]**	0.8 [2]
5. RUN	0.0 [0]	4.5 [3]	9.0 [6]	0.0 [0]	**86.6 [58]**	0.0 [0]	6.0 [4]	4.5 [3]	1.5 [1]	**88.1 [59]**

Numbers represent: % [# of instances]
